# Fatigue behaviour of dental crowns made from a novel high-performance polymer PEKK

**DOI:** 10.1007/s00784-021-03797-9

**Published:** 2021-01-28

**Authors:** Anne Katzenbach, Istabrak Dörsam, Helmut Stark, Christoph Bourauel, Ludger Keilig

**Affiliations:** 1 Oral Technology, Dental School, University of Bonn, Welschnonnenstr. 17, 53111 Bonn, Germany; 2 Department of Prosthetic Dentistry, Preclinical Education and Materials Science, Dental School, Rheinische Friedrich-Wilhelms University, University of Bonn, Welschnonnenstr. 17, 53111 Bonn, Germany

**Keywords:** Dental crowns, Polyetherketoneketone, Polymer, Fatigue, EN ISO 14801:2007, Artificial teeth

## Abstract

**Objectives:**

The aim of this study was, firstly, to analyse the long-time fatigue behaviour of crowns constructed from a novel polyetherketoneketone (PEKK) polymer, using artificial prepared teeth. Secondly, to determine the effect of the material’s stiffness that used as an artificial prepared tooth on the fatigue life of the PEKK crowns in comparison to human prepared teeth.

**Methods:**

Veneered crowns with a PEKK framework were constructed on three different prepared teeth: artificial polymethyl methacrylate (PMMA) teeth, artificial CoCr teeth and extracted human teeth. As far as applicable, the loading protocol was based on EN ISO 14801:2007 for fatigue testing of dental implants. After initial static fracture tests on three specimens from each group, the remaining crowns were loaded with different force levels until fracture or until 2 × 10^6^ loading cycles were reached. The number of loading cycles until failure was recorded. Wöhler curves were created to display the fatigue limits.

**Results:**

Static fracture limits as well as fatigue limits differed for all three core materials. The static fracture tests resulted in fracture limits of 1200 (± 293) N for the PMMA group, 1330 (± 219) N for the CoCr group and 899 (± 96) N for the human tooth group. Fatigue limits of 770 N, 840 N and 720 N were determined for the PMMA group, CoCr group and human tooth group, respectively.

**Conclusions:**

The determined fatigue limit of above 720 N (depending on the core material) is sufficiently high and a good performance of this crown material is expected in the clinical loading life. The results showed that using artificial teeth instead of natural teeth for fatigue testing of crowns might result in an overestimation of the fatigue limits of the crown material.

**Clinical relevance:**

PEKK-made crowns offer a stable and priceworthy treatment for patients, in particular those that suffer from metal allergy.

## Introduction

Metal and ceramic are commonly available restoration materials for single dental crown. It has proven to be a good choice to match the aesthetic and functional requirements of the patient and is considered by many dentists as a gold standard [1–4]. Nonetheless, this kind of restoration has some disadvantages: Besides the possible chipping of the ceramic facing, there is an aesthetic problem of the visible marginal metal line not covered by the facing material. Moreover, the heat treatment to establish a chemical bond between metal framework and veneering may alter the corrosive behaviour of the metal [5], as well as non-precious components may be added to the alloy to improve connection between the metal framework and the ceramics. The contact of saliva with the metal framework can lead to corrosive processes that result in a release of ions of the non-precious components. Most such ions are released in the region of the crown margin, directly adjacent to the gingival. This can be correlated to allergic reactions or a pathogenesis of periodontal diseases [5–7].

The disadvantages of metal restorations can be avoided using full ceramic restorations. One advantage of the recent CAD/CAM ceramics such as zirconia is reduced treatment costs and treatment time [8]. Another advantage is the improved aesthetics with the full ceramic reconstructions as compared to metal-ceramic reconstructions.

In the last years, different high-performance polymers have been introduced to different medical fields. Most of these polymers belong to the polyaryletherketone (PAEK) family in general or more specific to the polyetheretherketone (PEEK) sub-family. Numerous studies documented the successful clinical performance of such polymers in orthopaedic and spine patients as well as in dental applications [9–16].

Such high-performance polymers are also of interest for the field of dentistry. Common polymers are currently only used in very few cases as a permanent fixed restoration [17–19] due to limited stability with regard to the high biting forces. Material parameters of high-performance polymers seem to be optimised with respect to Young’s modulus and fracture strength, and thus could help to overcome these limitations.

Recently, a novel polymer from the polyetherketoneketone (PEKK) family has been introduced for dental applications. In a clinical study [20], the biocompatibility, handling and plaque accumulation as well as patient’s satisfaction for PEKK-made long-term temporary crowns and bridges were investigated and compared to the standard CoCr material. It was observed in this study that temporary restorations made from PEKK and CoCr improve the periodontal therapy and oral hygiene of patients. However, PEKK temporary restorations have high aesthetical advantage over CoCr-made restorations [20].

In an in vitro study [21], the retention forces of PEKK-made secondary crowns were analysed in combination with primary crowns made of four different dental alloys (gold, CoCr, zirconium and PEKK). It was concluded that all four primary crown materials tested with a high-performance polymer PEKK as secondary crown reach acceptable forces (up to 30 N) for overdenture retention over a period of 10,000 wear cycles [21].

In order to estimate the mechanical performance of the crowns made from such materials during their lifetime in the oral environment, it is not sufficient to perform static loading tests but the cyclic loading of each crown has to be investigated since the cyclic loading can influence the long-term success of these crowns. Dynamic loading is essential to test dental restoration materials, as the dental restoration has to endure repeated loading during biting, swallowing and speaking.

When performing such tests, a special care must be taken for choosing a suitable test environment representing—as far as possible—the clinical situation. Since it is difficult to collect enough extracted human teeth on which the crowns can be tested, artificial teeth offer an alternative approach. Nonetheless, it is not clear in how far the material properties of the prepared teeth can influence the long-term behaviour of the crowns.

Hence, the aim of this study was twofold: firstly, to analyse the long-time fatigue behaviour of crowns constructed from PEKK using artificial prepared teeth and secondly, to determine the effect of the material’s stiffness that used as an artificial prepared tooth on the fatigue life of the PEKK crowns in comparison to human prepared teeth.

## Materials and methods

### Geometry of test specimens

In the optimal case, extracted human teeth would be used for such fatigue testing. Unfortunately, in most cases, the same reasons that cause a tooth to be extracted are at the same time reasons why these extracted teeth are not suitable for fatigue testing. If a tooth defect can be repaired by creating a dental crown, it should not be extracted. Wisdom teeth are typically smaller than the first and second molars, they are sometimes not fully erupted and they are often damaged during extraction. Therefore, it is difficult to obtain a reasonable high number of human molar teeth in a sufficiently good quality with similar geometry. For this reason, three different test series were performed with different approaches to simulate the prepared teeth:

- Group 1 used 14 identical artificial teeth made from polymethyl methacrylate (PMMA).
- Group 2 used 11 identical artificial teeth made from a cobalt-chrome (CoCr) alloy.
- Group 3 used 9 extracted human wisdom teeth. The prepared human teeth varied in size and geometry. The occlusal surfaces of these teeth were individually reduced according to clinical practice to hold the crown.

The choice for the two materials for the artificial teeth—PMMA and CoCr—was done to have one material that is softer than the natural tooth as well as one material that is harder than the natural tooth. This was done to avoid that an unfortunate choice for a material distorts the fatigue testing results.

### Preparation of the test specimen

For all three groups, dental crowns made from PEKK (Pekkton® ivory, Cendres + Métaux SA., Biel, Switzerland) were manufactured on the prepared teeth according to clinical practice while maintaining the manufacturer’s instructions for use. As no local dental lab was available with the required equipment for processing Pekkton, the crowns were manufactured directly at the company Cendres + Métaux SA.

#### Crowns on artificial teeth

**Fig. 1:**
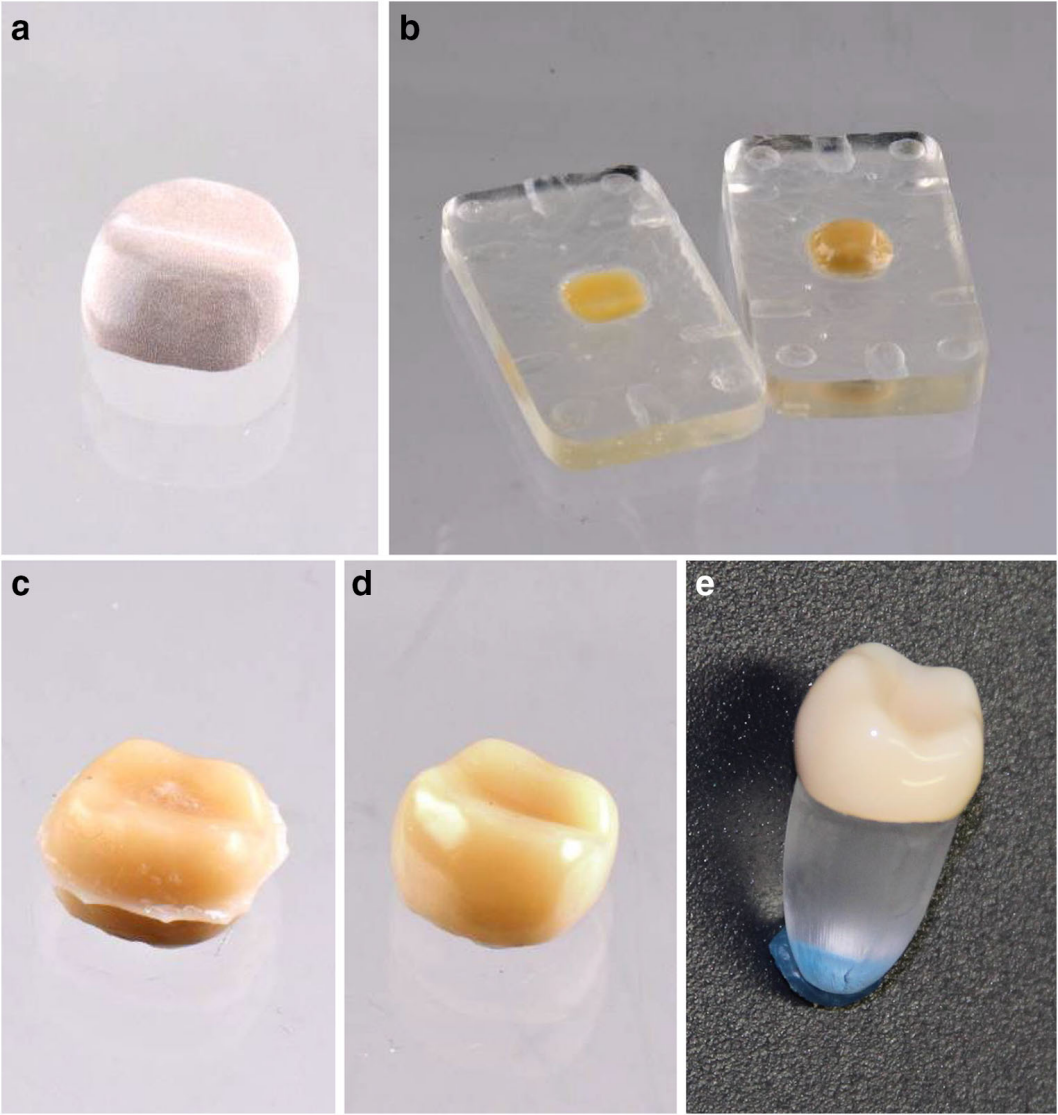
Preparation of the specimen for the identical crowns on artificial PMMA teeth. **a** Crown framework made from PEKK. **b** Two-part resin mould for the veneering. **c** Veneering directly after moulding. **d** Final crown with polished veneering. **e** Crown cemented on a PMMA tooth. Preparation of the specimens on CoCr teeth followed the same procedure

The crown framework was milled in wax using small milling machine (Impression Dental “CAM 4-K2”, vhf camfacture AG, Germany) and then pressed in Pekkton® ivory (Press-i-dent 654, DEKEMA, Germany). The thickness of the framework was 0.8 mm on the occlusal plane, and a wall thickness of at least 0.6 mm in all other areas (Fig. 1a). The crown frameworks were sandblasted with 110 μm Al_2_O_3_ at a pressure of 2 bar and then cleansed with oil-free pressurised air.

To archive an identical veneering for all specimens on the artificial teeth, a two-part resin mould was used (Fig. 1b). The mould was isolated twice with an artificial separator (Ivoclar Vivadent, Liechtenstein, Germany) prior to the application of the dentine paste. The layer thickness of the veneering was about 1.5 mm. For the veneering, only dentine body paste has been used. The use of incisal paste was not relevant for this study.

Priming of the surfaces was performed with SR Link (Ivoclar Vivadent, Liechtenstein, Germany). The primer was applied with a residence time of 2 to 3 min. Subsequently, Nexco Opaquer (Ivoclar Vivadent, Liechtenstein, Germany) was applied in 2 thin layers to the framework. The first layer was light-cured for 20 s for each segment (S.Light Jr., Wegold, Germany). The final hardening was performed with Solidilite EX (Shofu, Japan) for 3 min. After the final hardening, the inhibition layer was removed with a disposable sponge.

The framework was positioned in the mould and covered carefully with Nexco dentine paste (Ivoclar Vivadent, Liechtenstein, Germany; Fig. 1b). Both parts of the form were assembled by applying a light pressure. Intermediate hardening was performed for 1 min again with Solidilite EX. The crowns were removed from the mould (Fig. 1c, d) and completely coated with SR Gel (Ivoclar Vivadent, Liechtenstein, Germany). As a last step, the final polymerisation is performed using light-curing for 5 min. After moulding of the veneering, possible excess material as well as the edge was reworked using a mill. Polishing was performed by pre-polished with rubber wheels and finalising with a soft Bison-hair brush and Legabrill Diamond grinding paste (Cendres + Métaux SA, Switzerland). The final crown with veneering is shown in Fig. 1d, while Fig. 1e shows the crown cemented on the artificial PMMA molar.

The inner surfaces of the crowns were conditioned using Rocatec Pre and subsequently Rocatec Plus (3M, USA). Salinisation of the surfaces was performed with Monobond Plus (Ivoclar Vivadent, Liechtenstein, Germany) and primed with Luxatemp glaze&bond (DMG Chemisch-Pharmazeutische Fabrik GmbH, Germany). The abutments of the PMMA teeth were prepared by sandblasting using 110-μm abrasive blast media at a pressure of 1 bar. After the conditioning, the crowns are cemented on top of the PMMA teeth using Multilink Automix (Ivoclar Vivadent, Liechtenstein, Germany).

All specimens on artificial teeth (consisting of the tooth, the PEKK crown and the veneering) were prepared by a dental technician at Cendres + Métaux SA and then sent to the Materials Testing Lab of the Department of Oral Technology at the University of Bonn.

**Fig. 2:**
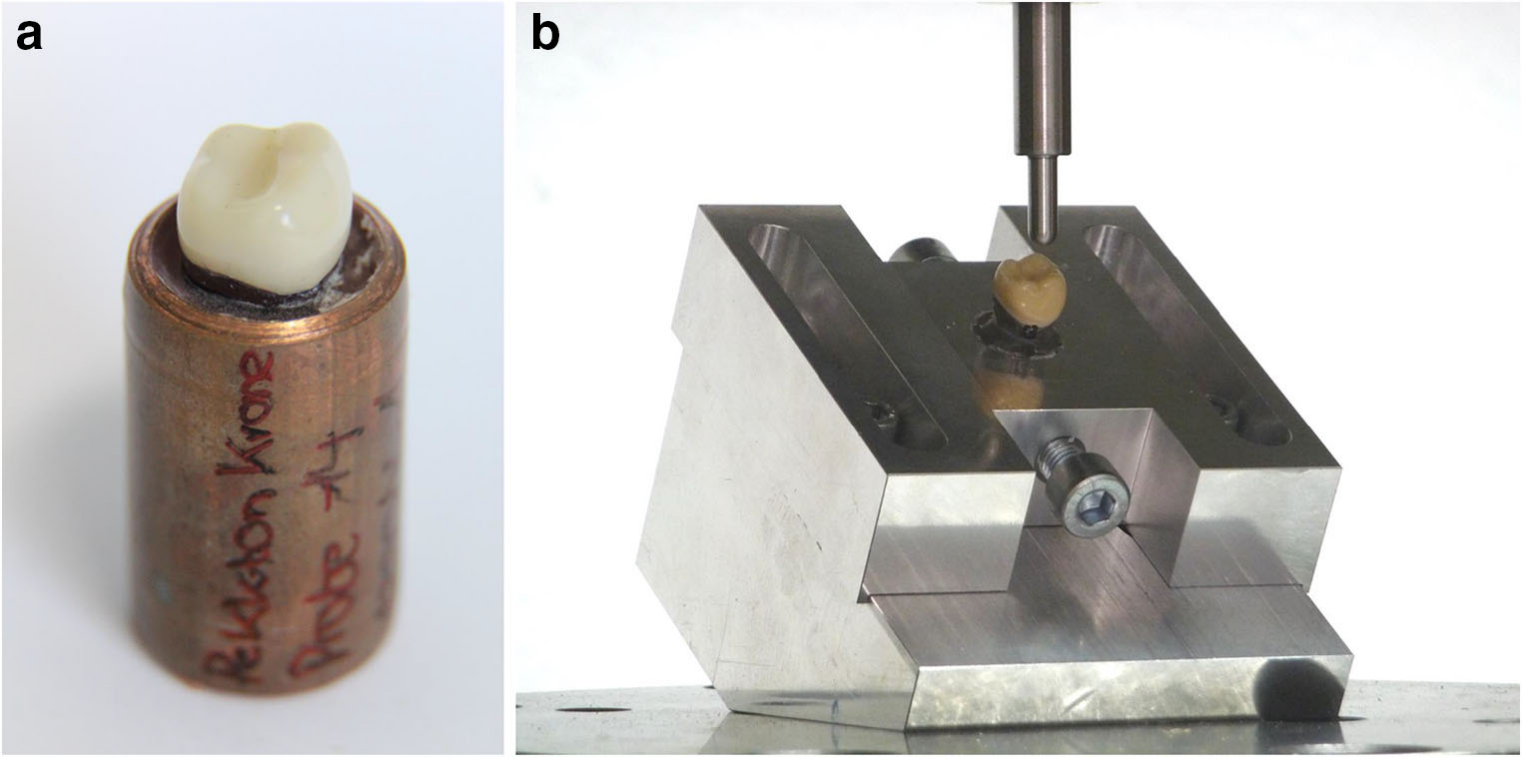
Specimen after embedding in a short copper tube (**a**) and inserted into the specimen holder (**b**)

For the fatigue testing, the specimens were embedded in a short piece of copper tube using resin (Pala-Xpress, Heraeus Kulzer GmbH, Germany) with a Young’s modulus of 3.0 GPa. According to the clinical situation, the specimens were embedded at the height of the cementoenamel junction (Fig. 2a). The copper tube with the specimen in turn was placed in a specimen holder for the permanent loading tests, which consisted of a block of aluminium with a central bore with a diameter of 18 mm to hold the copper tube with the inserted specimen (Fig. 2b). A list of all materials is compiled in Table 1.

**Table 1:** List of all materials used for manufacturing of the specimens

Material	Brand name	Manufacturer
PEKK^a^	Pekkton® ivory	Cendres + Métaux SA., Biel, Switzerland
PMMA^b^	ZENOTEC PMMA cast Disc	Wieland Dental + Technik GmbH, Pforzheim, Germany
CoCr	Remanium 2000 CoCrMoW	Dentaurum, Ispringen, Germany
Artificial separator	Separating Fluid	Ivoclar Vivadent, Liechtenstein, Germany
Primer	SR Link	Ivoclar Vivadent, Liechtenstein, Germany
Lab composites	Nexco Opaquer	Ivoclar Vivadent, Liechtenstein, Germany
Lab composites	Nexco Dentine	Ivoclar Vivadent, Liechtenstein, Germany
Glycerine-based masking gel	SR Gel	Ivoclar Vivadent, Liechtenstein, Germany
Saline	Monobond Plus	Ivoclar Vivadent, Liechtenstein, Germany
Primer	Luxatemp glaze&bond	DMG Chemisch-Pharmazeutische Fabrik GmbH, Germany
Resin	Pala-Xpress	Heraeus Kulzer GmbH, Germany
Conditioner	Rocatec Pre/Rocatec Plus	3M, USA

^a^*PEKK*, polyetherketoneketone

^b^*PMMA*, polymethyl methacrylate

#### Crowns on extracted natural teeth

A total of 9 extracted wisdom teeth were collected at a dental practice in Wesseling, Germany. All teeth were controlled to have no cracks and were suitable to be prepared to receive a crown. After extraction, they were disinfected in sodium azide solution. After disinfection, any remaining soft tissue was removed from the roots, and the teeth were stored in purified water at 8 °C for further use. The teeth were returned to this storage in between all the following steps. In order to aid the preparation of a crown, the teeth were embedded in short copper tubes in the same way as performed for the artificial specimens.

The preparation of the teeth was performed using coarse and fine diamonds according to common clinical practice at 40,000 rpm and with water cooling. Plaster replicas of these prepared crowns were created and send to Cendres + Métaux for the manufacture of the crowns. The creation of the crowns followed the same steps as described above. The only difference to the above specimens was that no mould was used, as each of the generated crowns followed the individual geometry of each individual tooth. The crowns were sent back to the Department of Prosthetic Dentistry, Preclinical Education and Materials Science and cemented on top of the teeth.

For this purpose, the teeth were cleansed with water, dried using pressurised oil-free air, and Multilink Automix Primer A+B (Ivoclar Vivadent, Schaan, Liechtenstein, Germany) was used as primer. All the following steps were performed in accordance with the crowns on artificial tooth.

### Procedure of the fatigue test

**Fig. 3:**
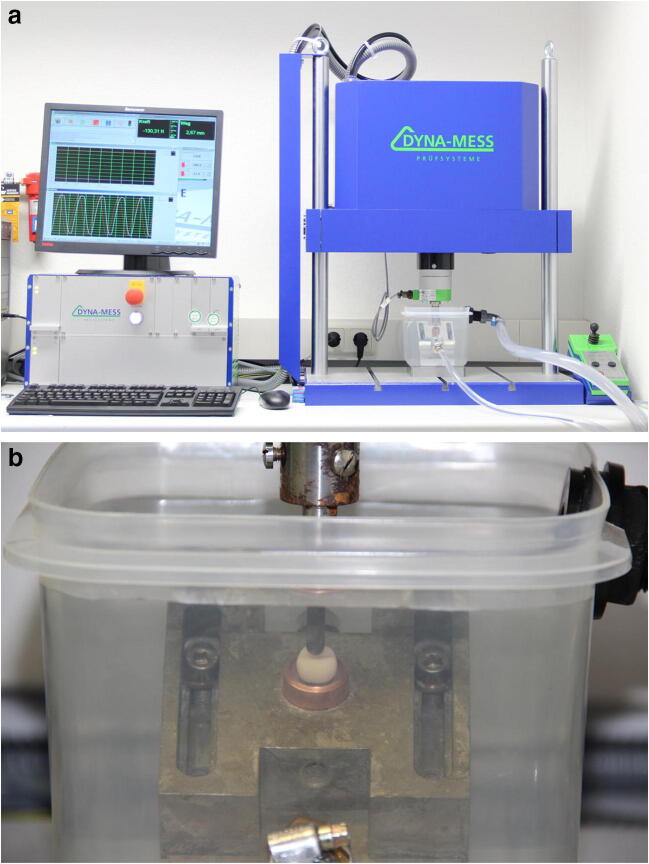
Materials testing setup “Dyna-Mess TP 5kN HF” used for the fatigue tests. Overview (**a**) and detailed view (**b**) of the basin used to realise the wet environment with a temperature of 37 ± 2 °C

The fatigue tests of the PEKK crowns were performed on a commercial pneumatic setup (“Dyna-Mess TP 5kN HF”, DYNA-MESS Prüfsystem GmbH, Germany, Fig. 3a) which fulfilled the requirements stated in EN ISO 14801:2007 (paragraph 5.1) [22]. The standard requires that specimen containing polymers must be tested in a fluid with a temperature of 37(± 2) °C. Force should be applied at an angle of 30° to the specimen’s long axis. These requirements were also transferred to the current tests for all three series of specimens. The copper tubes with the specimens were placed in a specimen holder which was mounted in an angle of 30° inside a small basin, which was connected to a tempered reservoir of purified water using flexible tubes (Fig. 3b). The tempered purified water was constantly pumped from the reservoir to the basin; overflowing fluid flows back through a second flexible tube.

The specimens were embedded up to the height of the cementoenamel junction according to the clinical situation around natural teeth, and load was applied directly on the centre of the crown using a semi-spherical indenter with a radius of 2.5 mm.

For each group, the fracture limit of these specimens was determined in static fraction tests using a commercial materials testing machine (ZMART.PRO, Zwick GmbH & Co. KG, Ulm, Germany), and the initial force level for fatigue testing was chosen as 80% of these fracture limits as required by EN ISO 14801:2007.

For tests in wet environment, the standard requires a loading frequency of 2 Hz and a total of 2 × 10^6^ loading cycles for each specimen. These parameters were chosen for the fatigue testing of the PEKK crowns as well.

### Failure criteria for the fatigue test

For all tests, a specimen was considered to have failed fatigue testing if either a fracture occurred in the veneering and/or the framework, or a crack was visible in the veneering and/or the framework. Due to the punctual load application, indentions at the point of force application directly beneath the tip of the thrust die were to be expected. Such an indention or small cracks that did not exceed the size of the indenter were not considered as failure, as long as no crack formation could be observed outside of this location. For all tests, a specimen was excluded from testing, if a fracture of the natural or artificial tooth occurred, and the crown did not fail by the above-mentioned criteria.

### Statistical analysis

Based on the data gained from the fatigue tests for all three groups, Wöhler curves were generated according to EN ISO 14801:2007. The number of specimens per group and force level was not high enough to perform a statistical analysis to determine possible significant differences among the three groups.

For the statistical analysis, a linear regression analysis was performed using the linearised data gained from plotting *log* (number of loading cycles) versus the corresponding force level [23]. The slopes of these regression lines as well as the associated 95% confidence intervals were determined and compared using Microsoft Excel 2010.

## Results

### Static fracture tests

The static fracture tests resulted in fracture limits of 1200 (± 293) N for the PMMA, 1330 (± 219) N for the CoCr and 899 (± 96) N for the human tooth groups, respectively. It was decided to use the same force levels for all three groups to allow a comparison between all three groups. An initial value of 1200 N was chosen for fatigue loading with the artificial teeth, and for each following force level, the force was decreased by 150 N.

### Fatigue tests

**Fig. 4:**
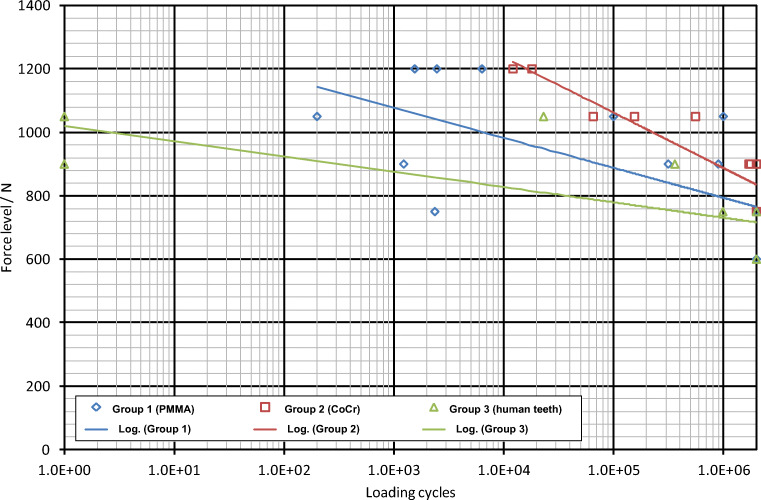
Wöhler curves as determined in the fatigue tests for the three groups of prepared teeth. Please note that symbols for successful specimens are identical and thus might represent up to 3 specimens withstanding 2 Mio cycles

All specimens of the group 1 and group 3 that were submitted to a load of 600 N survived during the fatigue test. For group 2 (CoCr), all specimens that were submitted to a load of 750 N survived. A summary of the performance of the different specimens in the groups can be seen in Table 2. The Wöhler curves (Fig. 4) showed fatigue limits of 770 N, 840 N and 720 N for groups 1, 2 and 3, respectively. The following section lists the results for each of the groups separately.

**Table 2:** Performance of the tested specimens in all three groups. The number of successfully completed tests, the number of failed tests due to cracks in the veneering, the number of failed tests due to a fracture of the veneering, and the number of failed tests due to a full fracture of the crown are listed for each group and each force level

Force level (N)	Group 1 (success/crack/veneering fracture/full fracture)	Group 2 (success/crack/veneering fracture/full fracture)	Group 3 (success/crack/veneering fracture/full fracture)
1200	0/2/0/1	0/0/0/2	-/-/-/-
1050	0/2/0/1	0/3/0/0	0/0/1/1
900	0/2/0/1	1/2/0/0	0/1/0/1
750	2/0/0/1	3/0/0/0	1/0/1/0
600	3/0/0/0	-/-/-/-	3/0/0/0

#### Artificial teeth made from PMMA (group 1)

**Fig. 5:**
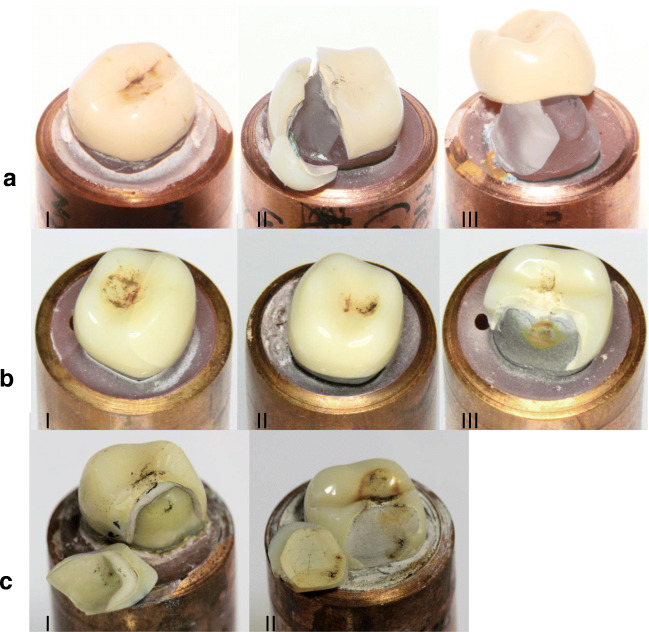
**a** Failure modes of crowns from the PMMA group. (I) Crack formation was detected approx. 900,000 cycles at 900 N. (II) Fracture of veneering and framework (900 N, 1231 cycles). (III) Fracture of the PMMA tooth and complete de-cementation of the otherwise undamaged crown (750 N, 3693 cycles, specimen was excluded and replaced). **b** Failure modes of crowns from the CoCr group. (I) Specimen after surviving 2 × 10^6^ fatigue cycles at 900 N. The dark marks at the centre of the crown are wear traces of direct contact to the indenter and were not considered as failure. (II) Crack in the veneering detected after 159,479 loading cycles at 1050 N. (III) Fracture of the veneering and the framework (18,103 cycles, 1200 N). **c** Failure modes of crowns on human teeth. (I) Specimen with a full fracture of the crown (veneering and framework) which occurred during the initial phase of the fatigue loading (900 N). (II) Fracture of the veneering with a part of the framework (990,186 cycles, 750 N)

A total of 15 specimens on artificial PMMA teeth were used in the fatigue tests. Those crowns on the PMMA teeth that failed in the fatigue testing showed a mixture of cracks in the veneering and complete fractures of the crown (i.e. fracture of veneering and framework, Fig. 5).

One specimen from this group had to be excluded, as the PMMA tooth fractured after only 3693 loading cycles at a force level of 750 N. This specimen was replaced, and the fatigue testing was repeated with the new specimen. A second crown had to be excluded and replaced, as the whole crown (framework with veneering) detached from the PMMA tooth after less than 10 cycles with a force level of 1200 N. The early failure might be an indication for a failure in the application of the cement. As the adhesion of the cement to the PMMA differs from the adhesion to natural teeth, this event was not considered as a failure of the crown.

#### Artificial teeth made from CoCr (group 2)

A total of 11 specimens on artificial CoCr teeth were submitted to the fatigue tests. Those crowns on the CoCr teeth that failed in the fatigue testing showed fractures of the whole crown only at a force level of 1200 N. For lower force levels, specimens failed only because of cracks visible in the veneering. No specimen on CoCr teeth had to be excluded due to a fracture of the artificial tooth.

#### Extracted human teeth (group 3)

A total of 9 specimens on human teeth were used in the fatigue tests. Due to the limited number of available teeth, only two specimens were used in each but the final force level. No crowns on human teeth at all were tested at a force level of 1200 N due to the limited number of available teeth as well as due to the lower static fracture limit of these specimens.

Those crowns on human prepared teeth that failed in the fatigue test showed mainly fractured crowns. Only in one case, at a force level of 900 N, a crack formation in the veneering was observed, and fatigue loading of this specimen stopped. In two cases, the whole crown including the framework fractured (at 1050 N and 900 N). Both full fractures occurred during the initial phase while the setup was adjusting the load control parameters. The remaining two fractures only involved the veneering while the framework was still partially intact (at 1050 N and 750 N). Such a separate failure of the veneering was only observed for the specimens on human teeth.

One specimen had to be excluded as the tooth fractured within the first ten loading cycles with a force level of 600 N. The fractured tooth showed a noticeable enlarged pulp and in turn a rather thin-walled tooth, which might have caused the fracture. The fatigue testing was repeated with a new specimen.

For the statistical analysis, the following slopes and 95% confidence intervals were determined for the three materials: − 94.6 (± 67.4) for PMMA, − 174.3 (± 60.1) for CoCr and − 48.4 (± 43.9) for the human teeth. The determined slopes for both groups with artificial teeth lay above the 95% confidence interval of the data for the crowns on human teeth. Thus, it can be deduced that the fatigue behaviour of the specimens differs depending on the chosen material for the artificial tooth (Table 3).

**Table 3:** Mean number of cycles and confidence intervals at the fulcrums of the Wöhler curves from Fig. 4

	Group 1, PMMA^a^		Group 2, CoCr		Group 3, human tooth	
Force in N	Mean no. of cycles	Confidence interval	Mean no. of cycles	Confidence interval	Mean no. of cycles	Confidence interval
600	2,000,000	0			2,000,000	0
750	1,334,121	1,305,098	2,000,000	0	1,495,000	808,153
900	405,410	516,178	1,829,727	170,552	180,956	289,582
1050	366,733	623,153	259,410	296,583	11,545	18,473
1200	3455	2884	15,140	4740		

^a^*PMMA*, polymethyl methacrylate

## Discussion

This study analysed the long-time fatigue behaviour of PEKK crowns on artificial prepared teeth and determined the effect of the material’s stiffness of the artificial prepared tooth on the fatigue life of the PEKK crowns in comparison to human prepared teeth.

Moreover, the influence of other parameters, namely, the bonding material and bonding technique, on the fracture and failure behaviour of the dental crowns was investigated.

In general, there are few studies about the bonding behaviour of the PAEK groups and most of these in vitro studies investigated the bonding behaviour of PEEK in combination with a bonding composite [24–27]. In addition, there are only few studies evaluating the effect of surface pre-treatment on the wettability and surface roughness of the PEKK surface. One study [28] investigated the shear bond strength composite to PEKK materials with three different surface treatments: chemical (95% sulfuric acid etching) and mechanical (airborne abrasion with 50 μm alumina and airborne abrasion with 110 μm silica-coating alumina) methods that are significantly effective to PEEK in the previous studies, and five bonding materials (Luxatemp glaze&bond, Visio.link, All-Bond Universal, Single Bond Universal, and Monobond Plus with Heliobond). This study concluded that the mechanical surface treatment behaves better with PEKK than the chemical one. In addition, self-etching universal adhesive (Single Bond Universal) can be an alternative bonding material to PEKK regardless of surface treatment method [28].

In our study, the inner surfaces of the crowns were chemically prepared. The inner surfaces were conditioned using Rocatec Pre and subsequently Rocatec Plus, salinised using Monobond Plus and primed with Luxatemp glaze&bond. The abutments of the PMMA teeth were prepared by sandblasting using 110-μm abrasive blast media at a pressure of 1 bar.

Since there is no suitable standardised test method for determining the fatigue limit of dental crowns [29–31], all tests in this study were performed—as far as applicable—in accordance with the international standard EN ISO 14801:2007 which lists all environmental conditions, requirements and parameters for a dynamic fatigue testing of endosseous dental implants with straight or angled abutments. Typically 5 fulcrums of the Wöhler curve have to be determined using a maximum of 3 specimens per force level. Depending on the number of successful loading cycles of the specimen at a given force level, the number of specimens per fulcrum, i.e. per force level, may be reduced to 2. This holds except for the lowest force level tested, as the stability criterion in ISO 14801:2007 reads that a minimum of 3 specimens have to withstand a certain force level, which then is called the fatigue limit. As is obvious from Fig. 4, crowns on human teeth already failed at the first cycle with forces of 900 and 1050 N, respectively, and thus testing at 1200 N was not reasonable. Consequently, the number of specimens on human teeth was reduced to a total of 9 specimens. The number of specimens in group 2 (CoCr) could be reduced as well, as 3 specimens survived 2 Mio loading cycles already at a force of 750 N.

In this study, the embedding height of the specimen was changed, and ongoing bone atrophy was not considered, as this is clinically not realistic for teeth. Additionally, a change of the height of the embedding would more probably increase the fatigue loading of the natural or artificial tooth than to change the fatigue loading of the crown. For implants tested according to this standard, Karl and Kelly [32] showed that the slowest specified test frequency of 2 Hz increases the likelihood of specimen failure, compared to an increased test frequency of 30 Hz [32]. While Lee et al. [33] showed that a dry or wet test environment does not influence the failure probability for implants, however, we still used the wet environment as this is mandatory according to EN ISO 14801:2007 due to the polymer components of the crowns.

The functional load is higher in the molar region than in the anterior region. Additionally, polymer crowns often have shortcomings in their aesthetic appearance. For this reason, it can be expected that they will be used more often in the posterior region, while other materials for crown restorations will dominate the anterior region. Accordingly, it was decided to perform the fatigue tests on molar teeth.

The presented fatigue tests of veneered dental crowns with PEKK framework showed different fatigue levels depending on the material of the prepared tooth. The specimens tested on natural teeth showed the lowest fatigue limit. The fatigue limits of the specimens on artificial teeth in general showed higher fatigue limits than on the human extracted teeth (dentin about 20,000 MPa and enamel about 65,000 MPa). PMMA has a stiffness of 3300 MPa (which is lower than natural teeth), while CoCr has a stiffness of 250,000 MPa (which is much stiffer than natural teeth). Thus, the fatigue testing on artificial teeth seems to overestimate the fatigue limit of the tested veneered polymer crowns. Nonetheless, it must be kept in mind that only a limited number of natural teeth of the necessary quality are available for fatigue testing. A fracture mode that only involved the veneering while the framework was partially intact could only be observed for the specimens on human teeth. This might be related to the fact that the cement used for fixing the crowns on the abutments is chosen for the best adhesion between tooth and framework, and its adhesive properties might differ on the artificial teeth made from PMMA or CoCr.

Nicolaisen et al. [3] performed similar fatigue tests on metal-ceramic and all-ceramic crowns on artificial teeth made from elephant tusk [3]. This material can easily be cut to form artificial teeth. As it is similar to natural teeth, the performance of the cement can more easily be compared to the clinical practice than the case with PMMA or CoCr teeth in this study. In their setup, the force was applied parallel to the tooth axis, and the force was increased stepwise (a force of 400 N over 600,000 cycles, followed by 200,000 cycles each at forces of 600, 800 and 1000 N). For both groups, early failures on a force level of 400 N were observed for some specimens.

In a recent study of Alsadon et al. [34], the fatigue behaviour of PEKK (Pekkton® ivory discs) was investigated. The prepared crowns were veneered with composite resin. The recorded fatigue limits for PEKK crowns was 790.4 ± 29.2 N. The study demonstrated high survival cycles of 1,170,000 for PEKK samples [34].

Different criteria influence the magnitude of the biting force to be expected in the clinical situation. Age, sex, health, state of the dentition, the prevalence of dental restorations such as tooth- and implant-supported prostheses [35], stomatognathic parafunctional disorders (bruxism) and habits (clenching, or grinding) [36], and even the emotional state of the patient [28] can influence the magnitude of the biting force. Depending on these parameters, literature reports values for the inter-individual maximum biting force in a wide range from some ten to several hundred Newton. In 2000, Fontijn-Tekamp et al. [36] determined biting forces for different groups with varying age with and without dentures [37]. Mean forces were highest in the young group with full dentition (mean age 22.7 ± 1.5 years, mean force 398 ± 103 N) and were significantly lower in the older group with complete dentition (mean age 54.1 ± 6.4 years, mean force 292 ± 94 N). In all groups with dentures, these forces decreased significantly. Of course, the effects of biting at maximum force differ depending on whether the load is evenly distributed across the whole dental arch or concentrated on one single tooth. The latter is clearly the most critical situation with respect to the fatigue loading of single tooth restorations. Ogura et al. [38] measured biting forces in molars and found in a group of twelve healthy volunteers a mean biting force of 205 N and 220 N in the lower and the upper left first molar, respectively, with a large inter-individual variation ranging from 25 to 667 N [38].

Despite the high number of papers dealing with the maximum bite force, there is to our knowledge no source available for a suggested minimum fatigue limit for dental restorations. It can be assumed that not every intra-oral loading cycle reaches the maximum amount of biting force. Gibbs et al. [39] found in a group of twenty volunteers with full dentition that on average forces during swallowing and chewing were at 41% and 36% of the maximum bite force, respectively [39]. Combining this with the above-mentioned highest values of average maximum bite forces in healthy subjects as reported by Fontijn-Tekamp et al. [35], a typical average fatigue load in the clinical application can be expected to be below 200 N and thus reasonable far below the determined fatigue limit of the veneered PEKK crown that were investigated in this paper.

For our knowledge, there is only limited literature available about the determination of the fatigue limit for dental crowns. There is few literature available about the cyclic loading for dental crowns; for example, a review study by Elshiyab et al. 2017 [40] gives a comprehensive overview on the fatigue behaviour of ceramic crowns under cyclic loading. The main difference to our study is that most recent studies only investigate if the crowns survive the long-term cyclic loading (with or without additional thermal pre-conditioning or loading) on one force level (typically at sub-critical level, for example 250 N, Aboushelib 2013) [41], and sometimes additionally measure the fracture limit before and after cyclic loading. Contrary to this, in our study, we determined the fracture limit for crowns made from PEKK by successively reducing the force level in cyclic loading until the specimen survived testing.

A further study needs to be done to compare PEKK crowns with other crowns’ materials such as metal-ceramic and full ceramic crowns. This was a limitation of the present study.

## Conclusion

A high fatigue limit of 720 N was determined for PEKK crowns that could provide a good, expected performance of this crown material in the clinical loading life. The results showed that using artificial teeth instead of natural teeth for fatigue testing of crowns might result in an overestimation of the fatigue limits of the crown material.
